# Supplementary material for: Mechanisms governing the pioneering and redistribution capabilities of the non-classical pioneer PU.1
Source: Nat Commun. 2020 Jan 21;11:402. doi: 10.1038/s41467-019-13960-2 (PMC6972792; doi:10.1038/s41467-019-13960-2)
Supplement: Supplementary file 7 — Source data [file 41467_2019_13960_MOESM7_ESM.zip › Source_Data/FigureS2/FigureS2B_MotifScanOutput/KmeansCluster12/homerResults/motif23.similar.html]

motif23

## Information for motif23

C
G
T
A
T
G
C
A
A
G
T
C
A
G
T
C
T
C
G
A
G
A
T
C
T
G
C
A
C
T
G
A
A
C
G
T
A
G
C
T
G
A
C
T
A
T
G
C
T
A
G
C
C
A
G
T
  
Reverse Opposite:  

G
C
T
A
A
C
T
G
A
T
C
G
C
T
G
A
T
C
G
A
G
T
C
A
A
G
C
T
A
C
G
T
C
A
T
G
A
C
G
T
A
C
T
G
T
A
C
G
A
C
G
T
A
C
G
T
  

|  |  |
| --- | --- |
| p-value: | 1e-21 |
| log p-value: | -4.957e+01 |
| Information Content per bp: | 1.774 |
| Number of Target Sequences with motif | 26.0 |
| Percentage of Target Sequences with motif | 1.45% |
| Number of Background Sequences with motif | 31.7 |
| Percentage of Background Sequences with motif | 0.07% |
| Average Position of motif in Targets | 105.8 +/- 52.0bp |
| Average Position of motif in Background | 110.3 +/- 45.8bp |
| Strand Bias (log2 ratio + to - strand density) | -0.2 |
| Multiplicity (# of sites on avg that occur together) | 1.00 |
| Motif File: | file (matrix) reverse opposite |

### Similar de novo motifs found

|  |  |  |  |  |  |  |  |
| --- | --- | --- | --- | --- | --- | --- | --- |
| Rank | Match Score | Redundant Motif | P-value | log P-value | % of Targets | % of Background | Motif file |
| 1 | 0.807 | C T A G C T A G T C A G C T G A T G C A C A G T C G A T A C T G A G C T C T G A C A T G A C G T A G C T | 1e-19 | -45.908978 | 3.51% | 0.78% | motif file (matrix) |
